# Harnessing a Ti-based MOF for selective adsorption and visible-light-driven water remediation†

**DOI:** 10.1039/d4ta01967a

**Published:** 2024-06-04

**Authors:** Stephen Nagaraju Myakala, Magdalena Ladisich, Pablo Ayala, Hannah Rabl, Samar Batool, Michael S. Elsaesser, Alexey Cherevan, Dominik Eder

**Affiliations:** a Institute of Materials Chemistry, Division of Molecular Materials Chemistry, TU Wien Getreidemarkt 9/BC/02 1060 Vienna Austria alexey.cherevan@tuwien.ac.at dominik.eder@tuwien.ac.at; b Department of Chemistry and Physics of Materials, Paris-Lodron-University of Salzburg 5020 Salzburg Austria

## Abstract

In pursuit of universal access to clean water, photocatalytic water remediation using metal–organic frameworks (MOFs) emerges as a strong alternative to the current wastewater treatment methods. In this study, we explore a unique Ti-based MOF comprised of 2D secondary-building units (SBUs) connected *via* biphenyl dicarboxylic acid (H_2_bpdc) ligands – denoted as COK-47 – as a visible-light-driven photocatalyst for organic dye degradation. Synthesized *via* a recently developed microwave-assisted method, COK-47 exhibits high hydrolytic stability, demonstrates a strong dye uptake, and shows noteworthy dye-degradation performance under UV, visible, and solar light, outperforming benchmark TiO_2_ and MIL-125-Ti photocatalysts. Due to its nanocrystalline structure and surface termination with organic linkers, COK-47 exhibits selective degradation of cationic pollutants while remaining inert towards anionic dyes, thus highlighting its potential for selective oxidation reactions. Mechanistic studies reveal the involvement of superoxide radicals in the degradation process and emphasize the need to minimize the recombination of photogenerated electron–hole pairs to achieve optimal performance. Post-catalytic studies further confirm the high stability and reusability of COK-47, making it a promising photocatalyst for water purification, organic transformation, and water splitting reactions under visible light.

## Abstract 2

This study demonstrates the potential of a novel 2D-SBU COK-47-Ti MOF for visible-light-driven wastewater treatment through selective adsorption and photodegradation and shows its excellent performance superior to that of TiO_2_ and MIL-125-Ti.

## Graphical abstract

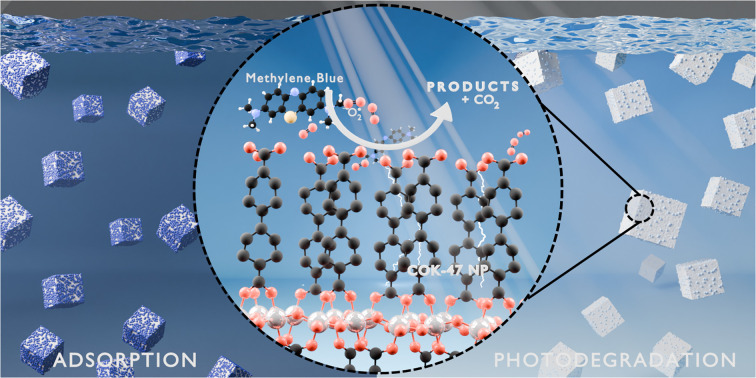

## Introduction

In 2017, the UN put into motion the Sustainable Development Goal 6 (SDG6) which aims to ensure access to clean water for all by 2030.^1^ Wastewater recycling, a strong pillar of sustainable water management, appears as an attractive strategy to achieve this goal in tackling the shortage of clean freshwater in many countries. As of today, almost 20% of the wastewater is polluted with colorant dye molecules from the fabrics used in textile industries.^2,3^ Additionally, a large number of these commercially applied synthetic dyes such as methylene blue (MB) and crystal violet (CV) are deemed non-biodegradable and are extremely stable over long periods of time due to the presence of aromatic groups in their chemical structures.^4–6^ While largely harmless when used to color fabrics and textiles, excess amounts of such dyes discharged into large water bodies can be highly carcinogenic to aquatic life.^7^

Currently, the physical and biological wastewater treatment methods used for treatment strongly suffer from slow degradation rates and also produce unwanted by-products. On the other hand, advanced oxidation processes (AOPs), where *in situ* generated radicals (*e.g.* OH˙, O_2_˙^−^) are used to degrade organic contaminants, serve as a great alternative solution to them capitalizing on their fast degradation rates without any sludge production.^8^ While most common AOPs require the use of H_2_O_2_ and O_3_ as a source of such reactive oxygen species (ROS), producing these radicals directly from water *via* photocatalytic pathways can further ensure the sustainability of the AOPs and allow for efficient use of natural sunlight.^9,10^

Among a plethora of potential semiconductor materials capable of driving photocatalytic generation of ROS, metal–organic frameworks (MOFs), consisting of metal–oxo secondary-building units (SBUs) connected *via* organic linker molecules, are alluring due to their high surface areas, extended microporosity characteristics and robust structural and chemical tunability.^11,12^ While these properties render MOFs suitable for a wide range of catalytic applications, most MOF structures are strongly limited by their hydrolytic fragility when exposed to aqueous environments.^13–15^ In contrast, MOFs based on Ti- and Zr-containing SBUs – such as UiO-66 and MIL-125 families – have demonstrated some of the highest resistance towards hydrolysis, which facilitates their implementation for wastewater remediation technologies including those based on photocatalysis.^9,16,17^ Most recently, an ^18^advanced class of 1D and 2D SBU based MOFs exhibiting superior ability to separate and conduct photogenerated charge carriers has come into the light owing to the chain-like and planar SBUs, respectively.^19–22,31^

In 2019, Smolders *et al.* reported the first synthesis of a 2D-SBU based MOF, called COK-47, built of Ti–oxo SBUs connected *via* the biphenyl dicarboxylic acid (H_2_bpdc) ligand. In addition to a complete set of characterizations for the MOF structure, the authors also demonstrated its potential as a photocatalyst for rhodamine 6G degradation under UV illumination.^23^ Building on this, our group recently reported an optimized microwave-assisted synthesis of COK-47 resulting in isotropic nanometer-sized particle while preserving ideal ligand to metal stoichiometry,^18^ which afforded lower semiconducting bandgap enabling efficient utilization of visible light. We further demonstrated excellent charge carrier lifetimes by means of time-resolved emission spectroscopy (TRES) and showed COK-47 to be one of the best MOF-based photosystems for visible-light-driven hydrogen production.^18^ Following these results, herein we unravel the potential of, COK-47, towards the visible-light-driven photocatalytic oxidative degradation of organic dyes. We test its degradation capabilities over a range of cationic and anionic dyes such as methylene blue (MB), crystal violet (CV), toluidine blue (TB), and methyl orange (MO) (Fig. S1a–d†). We unravel and discuss selective degradation nature of COK-47 towards cationic dyes specifically, which indicates its prospects towards selective oxidation reactions. Various radical scavenging experiments are performed to shed light on the MB degradation mechanism. Finally, we test the stability and recyclability of COK-47 after multiple degradation cycles, which demonstrate its potential for visible-light-driven photocatalytic water remediation.

## Results and discussion

### Synthesis and characterization

COK-47 was prepared based on the microwave-assisted synthesis recently reported by our group.^18^ Its structure, composition, and optoelectronic properties were characterized using a variety of techniques including X-ray diffraction (XRD), Fourier transform infrared (FTIR), transmission electron microscopy (TEM), and diffuse reflectance spectroscopy (DRS).

**Fig. 1:**
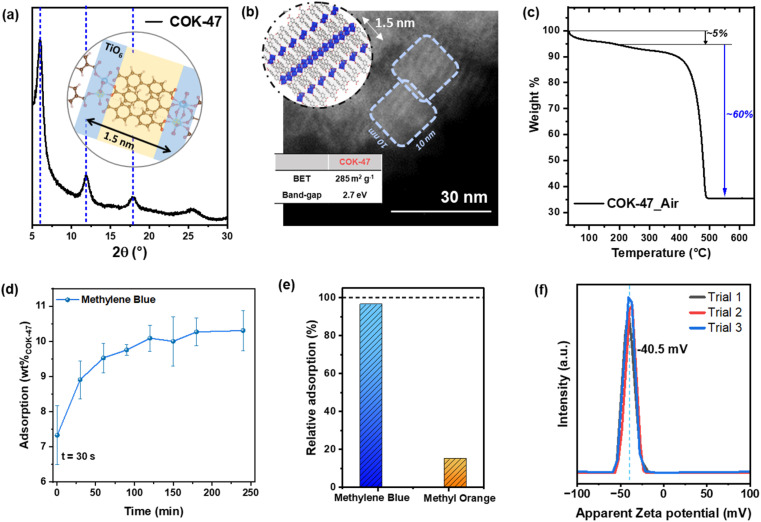
(a) XRD pattern of pristine COK-47 with blue lines representing the reference peak positions from literature (inset: model structure showing the distance between two SBU sheets connected *via* bpdc^2−^ linkers). (b) HR-TEM image of an aggregate of COK-47 particles where the bright lines show the 2D-SBU layers spaced evenly at a distance of 1.5 nm, with an overall particle dimension of 10 × 10 nm^2^ (inset schematic sketch of a model COK-47 nanoparticle with the blue octahedrons representing the 2D-SBU layers connected *via* the bpdc^2−^ linker molecules; and table showing the specific surface area (SSA) and optical band gap of pristine COK-47). (c) TGA curve of pristine COK-47 showing the overall weight loss due to ligand decomposition in synthetic air atmosphere. (d) MB adsorption profile with respect to weight of adsorbent (averaged over 8 experiments), measured under dark conditions over the course of 4 h. (e) Relative dye adsorption (in the dark) when using COK-47 in a mixture of methylene blue and methyl orange solutions (6 ppm each) after equilibration (120 min). (f) Zeta potential curves of pristine COK-47 powder finely dispersed in DI water as a solvent.

The XRD diffractogram (Fig. 1a) of the as-prepared COK-47 MOF features three characteristic peaks, with the first peak corresponding to the interlayer spacing of ∼1.5 nm, in line with the length of the bpdc^2−^ linker in the MOF structure (Fig. 1a inset). FTIR analysis reveals peaks that correspond to carboxylate groups (1572, 1506 and 1416 cm^−1^) along with the fingerprint region matching well with reported literature (Fig. S1e†).^18,23^ A representative TEM image of the as-prepared COK-47 powder in Fig. 1b shows a collection of closely packed nanocrystals (∼10 nm in size) and further corroborates a layered SBU structure of this MOF. The use of our microwave-assisted synthesis procedure allows controlling the growth to obtain uniform crystalline domains resulting in isotropic COK-47 nanoparticles, which is expected to deliver optimal adsorption and catalytic properties. Despite the intrinsic non-porous nature of our MOF, this nano-crystalline morphology results in a relatively high surface area material affording a surface area value of 285 m^2^ g^−1^.^18^ Importantly for its photoresponse in the visible-light range, this unique COK-47 morphology results in a red shift of the absorption edge maximum that narrows its bandgap to about 2.7 eV, which is beneficial for the prospected applications in photocatalysis (Fig. S1f†). The TGA curve (Fig. 1c) of the as prepared COK-47 show an initial solvent weight loss of ∼5 wt% (below 200 °C) followed by a weight loss of 60 wt%, with TiO_2_ (anatase – 04-022-3337) as the final oxidative product, verified *via* XRD analysis (Fig. S2a†). Interestingly, this weight loss is higher than the value calculated from its stoichiometric formula (∼58 wt%, ESI Note 1†), indicating the presence of excess ligands. Additionally, any weight loss at 400 °C – which would correspond to loss of any unwashed free H_2_bpdc – is completely absent from the TGA curve,^18^ which strongly suggests the excess ligands are present as a surface passivation to the MOF nanoparticle.^23^

### Adsorption characteristics

Generally, owing to the high surface areas available in the MOF, it is important to account for the initial dye adsorption on the catalyst to establish an optimum equilibration time required before the beginning of the photodegradation experiment. The concentration profile of MB in COK-47 recorded for a total duration of 4 h in the absence of light, reveals a strong adsorption for the first 90 min followed by saturation indicated by a constant level of MB concentration after 2 h (Fig. 1d). Interestingly, adsorption studies carried out using two adsorbent dosages (Fig. S2b†) reveal that using lower COK-47 concentration (0.1–0.2 mg mL^−1^) leads to an adsorption capacity value close to the theoretical maximum (ESI Note 2†), while concentrations higher than 0.2 mg mL^−1^ begin to show detrimental effects likely due to particle agglomeration. Based on these preliminary experiments in the dark, to achieve optimized adsorption performance, we fixed COK-47 amount to 10 mg (∼0.2 mg mL^−1^) and proceeded with 2 h of equilibration time sufficient to achieve adsorption equilibrium. Interestingly, upon examining the adsorption profiles of various dyes on COK-47, we observed a strong uptake of MB, CV, and TB – which adsorb up to 75% of the initial dye level – whereas only an insignificant amount of MO is taken up by an otherwise identical COK-47 suspension. In the representative case of MB, we note that most of the adsorption happens in the first minute of mixing, suggesting that there is a strong driving force for the dissolved dye molecules to interact with COK-47. Additionally, our adsorption studies reveal an uptake capacity of ∼100 mg g^−1^ (∼10 wt%), which ranks COK-47 as an intermediately strong adsorbent towards MB when compared to other MOFs (Table S1†), as expected from its moderate surface area. Additionally, the experimental value is in good agreement with the theoretically maximum (∼12 wt%) suggesting a monolayer dye adsorption model on the external surface of COK-47 nanoparticles (ESI Note 2†). This could be seen as an optimal scenario when considering MB degradation, since multilayer coverage of the MOF surface may result in blocking the absorption of light photons or inhibiting the generation of ROS thus leading to an inefficient photodegradation. The monolayer coverage suggested from experimental and theoretical calculations would additionally ensure effective diffusion of new MB molecules to the catalytic sites allowing its continuous degradation. To further explore the extent of the selective nature of COK-47, we carried out standard adsorption experiments in a solution containing the mixture of MB (cationic) and MO (anionic) dyes (details in Methods). Fig. 1e plots relative adsorption of both the dyes after equilibration and shows that COK-47 has a strong preference towards MB (Fig. S2c†). These results clearly demonstrate selectivity of COK-47 towards cationic dye pollutants present in wastewater.

To gain further insight into interactions involved in the dye adsorption process, we employed point-of-zero charge (PZC) and zeta potential measurements with the aim of identifying the isoelectric point and quantifying the surface charge of COK-47, respectively.^24–26^ PZC measurements using 0.1 M KNO_3_ solution reveal an isoelectric point at pH of 4.22 for pristine COK-47, implying a negatively charged MOF surface in solutions with a pH higher than 4 (Fig. S2d†). Complementarily, zeta potential measurements conducted at pH = 7 – that is relevant to the conditions of the adsorption and photocatalytic studies presented in this work – also show a negative surface charge of −40.55 mV (Fig. 1f).^27^ Both results demonstrate that the MOF surface exhibits a strong negative charge, which can be largely attributed to the presence of deprotonated carboxylic groups (COO^−^) terminating the MOF nanoparticle, in line with our TGA results. Considering the ionic nature of the dyes in question (MO is anionic, MB, CV, and TB are cationic), the results suggest that the MO anions are strongly repelled from the adsorbent's surface impeding their adsorption and any subsequent photodegradation (Fig. S3a†); conversely, the cationic nature of MB, CV, and TB facilitates them to be readily picked up by the MOF surface, which can later aid their efficient degradation. Therefore, we conclude that the initial adsorption of the dyes is primarily driven by the electrostatic interactions between COK-47 surface and the dye molecules, however, we don't exclude that the adsorption process can additionally benefit from π–π and hydrogen bonding interactions between the bpdc^2−^ ligands and MB molecules, as reported by Ali *et al.*^24^

### Photodegradation performance

#### Activity and benchmarking

Photodegradation experiments were carried out under both UV (280–400 nm) and visible (400–700 nm) light illumination. A set of reference experiments was first conducted with solution containing only dye molecules revealing no degradation in the absence of COK-47, *e.g.* due to photobleaching effects, thus confirming the importance the catalysts in the photocatalytic process (Fig. S3b and ESI Note 3†). The degradation performance of COK-47 was benchmarked internally against two relevant Ti-based photocatalysts including TiO_2_ (anatase and commercial P25) and MIL-125-Ti commonly used by the photocatalytic- and MOF-communities, respectively. As a figure of merit to compare the performance of each catalyst under various illumination conditions, in the following we discuss the values of overall removal efficiency and degradation rates (details in Methods).^28^

**Fig. 2:**
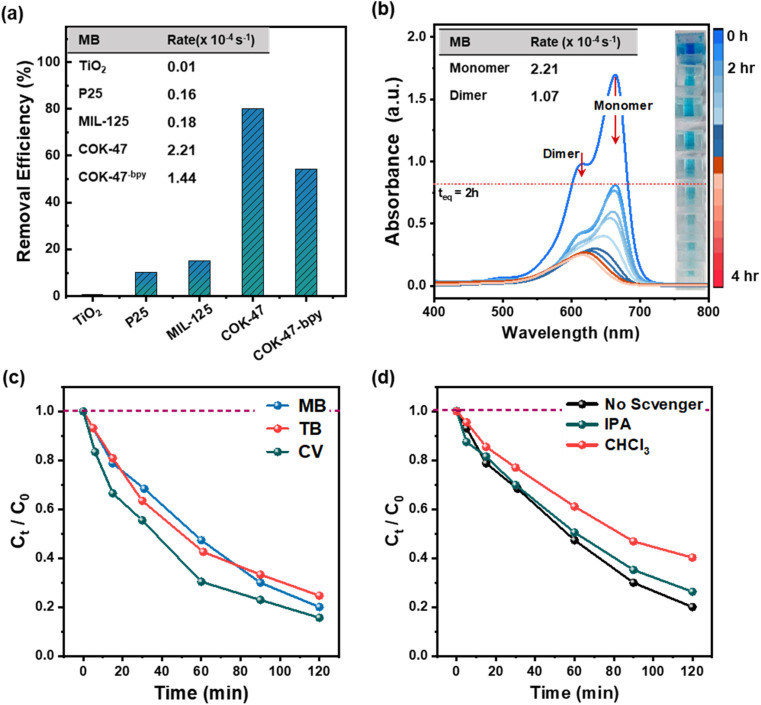
(a) Overall removal efficiency of methylene blue using different Ti based-catalysts under visible light irradiation (2 h). (b) UV-Vis spectrum of MB degradation over pristine COK-47 under visible light illumination, with blue and red arrows depicting the degradation of the monomeric and dimeric forms of MB, respectively (inset: table showing the corresponding degradation rates). (c) Degradation profiles of different cationic dyes (MB, CV, and TB) on pristine COK-47. (d) Degradation profile of MB on COK-47 in the presence of oxygen radical and OH radical scavengers *via* CHCl_3_, and IPA, respectively.

When exposed to UV irradiation, COK-47 shows an overall removal efficiency on par with anatase and P25 TiO_2_ (97.9% *vs.* 97.5% *vs.* 99.4%) which are considered two of the most promising UV-active photocatalysts for wastewater purification (Fig. S3e†).^29,30^ However, the advantage of COK-47 becomes evident under visible light. As presented in Fig. 2a, COK-47 demonstrates an unmatched removal efficiency of 80%, accompanied by record degradation rates reaching as high as 2.21 × 10^−4^ s^−1^ (Fig. 2b, inset). In contrast, MIL-125-Ti, P25 and anatase TiO_2_ only remove 18%, 10% and 1% of the dye, respectively, yielding significantly lower degradation rates of about 1.8 × 10^−5^, 1.6 × 10^−5^, and 0.1 × 10^−5^ s^−1^. The inset on the right in Fig. 2b provides additional visual representation of the degree of MB solution discoloration achieved with noble-metal-free COK-47 under 2 hours of visible-light illumination. Given the excellent performance demonstrated under both UV and visible light illumination, we also tested COK-47 in the presence of natural sunlight, emulating real-world conditions (see Methods). Here we observe an overall removal efficiency of ∼75% after 2 h under sunlight exposure with MB degradation profiles similar to those obtained under artificial visible-LED light (Fig. S3f, ESI Note 4†).

Transient photocurrent measurements shed more light on this photocatalytic behaviour with respect to the charge extraction ability of each catalyst (Fig. S3c†). Notably, pristine COK-47 generates an initial photocurrent response of over 3.5 μA reaching a stable value ∼1.3 μA overtime (∼36 times higher than MIL-125-Ti) under visible light illumination. While the large photocurrent gap under visible illumination can be attributed to the limited absorption of visible light by MIL-125-Ti, the strong enhancement in the latter can be attributed to the better electron mobility in the 2D SBU sheets of COK-47, in comparison to MIL-125-Ti. A similar photocurrent trend can be observed under UV light irradiation which could again be attributed to the better electron mobility in the 2D SBU channels of COK-47 (Fig. S3d†).^19,21,29^ The results signify the excellent performance of COK-47 regarding the photocatalytic degradation of MB with high adsorption and record degradation rates, especially under visible and sunlight conditions.

#### Pore accessibility and versatility

Fig. 2b shows the evolution of UV-Vis absorption profiles of MB upon visible light irradiation. The absorption maxima at 664 and 612 nm correspond to the presence of MB monomers (MB_1_) and dimers (MB_2_), respectively (Fig. S4a and b†).^32^ No absorption peaks corresponding to higher aggregates such as trimeric and tetrameric forms were observed for concentrations below 70 ppm.^33^

Re-calculating the rate constants based on this deconvolution, we observe a 2 times higher degradation rate for MB_1_ as compared to its dimer MB_2_, which explains the overall red shift caused in the absorption spectra. The fact that COK-47 degrades MB_1_ species quicker compared to MB_2_ can be related to their smaller size and better access to the micropores.

Next, following the strong preference of COK-47 to interact with positively charged dyes, Fig. 2c shows the photocatalytic performance of COK-47 on degrading other cationic dyes, such as CV and TB. Since these are metachromatic dyes,^34–36^ we observe a similar behaviour in their UV-Vis profile as for MB upon degradation, suggesting that the rate of degradation is higher for the monomeric form of the dye as compared to its dimeric analogue (Fig. S4c and d†). Importantly, in the case of CV and TB, we also observe generally analogous degradation profiles, removal efficiencies and compute similar degradation constants as compared to the ones observed for MB (2.2 × 10^−4^, 2.7 × 10^−4^ and 2.1 × 10^−4^ s^−1^, for MB, CV, and TB, respectively) (Table 1). This result hints at a common degradation mechanism for these dyes as we discuss in detail in the following section.

**Table 1:** Photocatalytic degradation rates and removal efficiencies of various cationic dyes using COK-47 under visible light illumination

Dye	Degradation rate (×10^−4^ s^−1^)	Removal efficiency (%)
MB	2.2	80
TB	2.1	75
CV	2.7	85

### Mechanistic studies

Photocatalytic experiments with widely used scavenging molecules, namely, triethanolamine (TEOA), benzoquinone (BQ), and isopropyl alcohol (IPA) were performed to gain insights into the possible degradation pathways of MB on COK-47. Each of these scavengers was used to probe the influence of a specific oxidative species, namely, hole (h^+^), superoxide radical (O_2_˙^−^), and hydroxyl radical (OH˙), on the extent of the photodegradation process.

Fig. S5a† shows the MB concentration profiles obtained during 2 h of visible light illumination in the presence of these scavengers. Interestingly, we observe a ∼1.5 and ∼2.4 fold increase in the degradation rates when MB-MOF suspension was irradiated in the presence of BQ and TEOA, respectively. These results could be explained by the generation of more reactive BQ* intermediates or by role of TEOA in enhancing the charge separation efficiency (ESI Note 5†), however, our reference experiments carried out in the absence of COK-47 also demonstrate that BQ- and TEOA-containing MB solutions undergo strong photochemical transformation when illuminated with light^37^ making these scavengers unsuitable for our investigations. In order to address this gap, we further explored chloroform (CHCl_3_) and disodium ethylenediaminetetraacetic acid (Na_2_EDTA) known for their ability to scavenge O_2_˙^−^ and h^+^ selectively.^38,39^ while CHCl_3_ and IPA did not show any direct interaction with MB under illumination making them suitable for our mechanistic studies, Na_2_EDTA was able to slowly degrade MB under visible light illumination (ESI Note 5†).^40,41^

Fig. 2d shows the MB degradation profiles with IPA and CH_3_Cl as scavengers. Contrary to the experiments with BQ, TEOA and Na_2_EDTA, we observe almost no change in the degradation rate in the presence of IPA – known for its ability to trap OH˙ radicals in the solution – which suggests that OH˙ generated at the MOF/solution interface play only a minor role in the overall degradation. In contrast to this, a strong decrease in the degradation (∼3.8 times lower) rate in the presence of CHCl_3_ is evident. Due to its low polarity, CHCl_3_ is largely immiscible with water, resulting in it forming a layer around the catalyst surface and scavenging O_2_˙^−^ radicals generated from the adsorbed O_2_. The strong drop of MB degradation in the presence of otherwise inert CHCl_3_ suggests that surface generated O_2_˙^−^ are the major ROS taking part in the process.

Overall, our scavenger experiments suggest that the prime mechanisms responsible in the light-driven oxidation of MB molecules by COK-47 involve the formation of ROS, such as superoxide radicals, however our data also points out that electron/hole separation efficiency can be one of the key points to further increase the photoactivity of COK-47. In order to test this hypothesis, we investigated a COK-47 analogue, namely COK-47-bpy, based on 2,2′-bipyridine-5,5′-dicarboxylic acid (H_2_bpydc) ligand. Despite having otherwise identical structure and connectivity, COK-47-bpy underperforms in the photocatalytic MB degradation by ∼35% compared to pristine COK-47 (Fig. 2a). Complementary transient photocurrent measurements also reveal a lower photocurrent response from COK-47-bpy in comparison to pristine COK-47 under both UV- and visible-light suggesting a weaker charge extraction ability in the presence of bpydc^2−^ ligands (ESI Note 6†). In line with this, time-resolved emission spectroscopy (TRES) measurements of COK-47-bpy confirm that the presence of –N– moiety on the ligand has a strong negative effect on the electron–hole recombination process as a ∼30% decrease in carrier lifetimes is recorded in comparison to COK-47 (Fig. S6a†). This facilitated charge recombination can be well correlated with the extend of lowered degradation rates of COK-47-bpy. Overall, these data support the hypothesis that charge extraction is key to the outstanding performance of COK-47: its biphenyl ligand allows for efficient ligand-to-metal charge transfer (LMCT), which avoids trapping of the electrons on the ligand and reduces electron/hole recombination rates;^42^ its 2D SBU nature further enables efficient directional transfer of the electrons to the reduction sites, while holes are able to take part in the oxidation reactions and effectively generate ROSs.

### Post-catalytic studies and recyclability

**Fig. 3:**
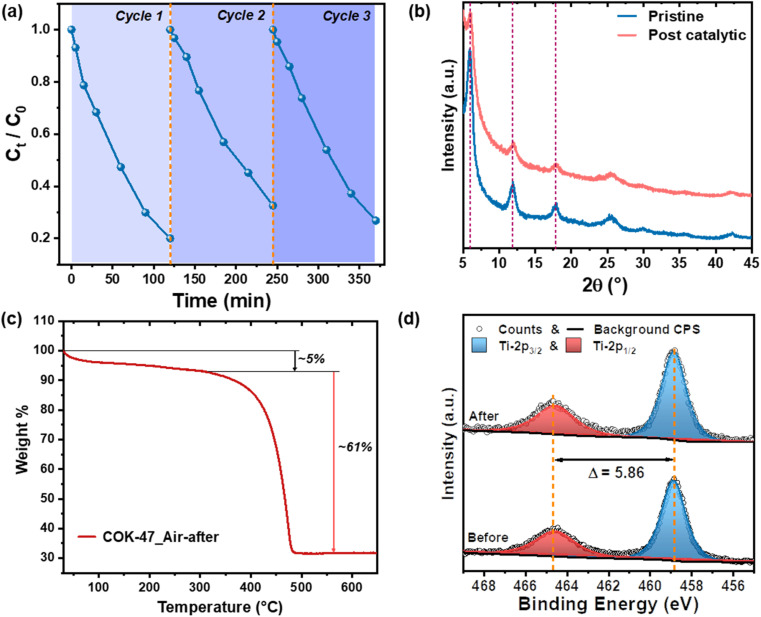
(a) MB concentration profiles for 3 cycles showing the recyclability and stability of COK-47 under reaction conditions. (b) XRD pattern of COK-47 before and after photocatalytic degradation of MB. (c) TGA curves showing weight loss of COK-47 after a single MB degradation cycle, in air and N_2_. (d) XPS spectra of the Ti 2p edge of COK-47 before and after multiple photocatalytic degradation reactions.

Fig. 3a shows the concentration profiles of MB during 3 photocatalytic degradation cycles using COK-47. Between each measurement, the MOF was recovered, dried and then suspended in a fresh MB solution for the subsequent cycle. COK-47 exhibits stable degradation rates over all 3 cycles, with a consistent photocatalytic removal efficiency of ∼80%. XRD analyses post-catalysis – over 10 hours of illumination – reveal the presence of all the characteristic COK-47 peaks indicating retained structural integrity of the MOF (Fig. 3b). Recalling the significance of chemical and hydrolytic stability of the MOF for the application in water purification, we also examined COK-47 following the dye saturation stage (before the photo-degradation process). XRD analysis shows no shifts in the diffraction pattern nor the appearance of any new peaks in the MB-saturated-COK-47, suggesting that its strong adsorption ability comes without any sacrifice in its crystallinity and overall structure (Fig. S7a†). Following this, complementary total reflection X-rays fluorescence (TXRF) and thermogravimetric measurements were carried out with post-catalytic MOFs to verify if any MOF underwent degradation resulting in potential leaching of Ti-ions and/or organic ligands into the solution took place. TXRF quantitatively shows the presence of only a negligible amount of Ti (0.003%) in the solution after multiple MB degradation cycles further confirming stability of COK-47. In line with this, TGA-derived weight loss of the MOF powder recovered after photocatalysis shows no change in the organic content of the MOF compared to that of the pristine COK-47 (Fig. 3c). This result corroborates that the main mechanism of dye removal is based on its photodegradation – as opposed to just adsorption-based removal – since any residual dye would result in a higher weight loss. Additional XPS measurements further show no changes in the Ti 2p edge profile even after multiple cycles of MB degradation (Fig. 3d). These datasets conclusively show high structural and compositional stability of COK-47 towards light-driven dye degradation and commend this MOFs for other light-driven water purification applications.

## Conclusion

We prepared and investigated COK-47 as a high-surface area, stable and efficient catalyst for photodegradation of various harmful dye pollutants. We observed a high adsorption capacity up to about 100 mg g^−1^ and a remarkable degradation performance under both UV and visible-light illumination, with an overall removal efficiency of 97% and 80%, respectively. We also explained its ability to selectively degrade cationic pollutant molecules such as methylene blue, while being completely repellent to anionic dyes like methyl orange, which we largely attribute to its negatively charged surface due to ligand passivation. Notably, mechanistic studies to unravel photodegradation pathway showed superoxide radicals to play a major role, while also highlighting the importance of low charge recombination in COK-47 that leads to its optimal performance. In this respect, the 2D nature of the framework's SBUs seems especially beneficial leading to high photocurrents, facilitated charge separation and directional transport of the electrons to the active sites. Furthermore, our comprehensive post-catalytic characterization and recyclability datasets conclusively show high structural and compositional stability of COK-47 towards visible-light-driven dye degradation, which commends this unique MOF for other applications where efficient charge separation and extraction is required, such as in water purification, organic transformation, and water splitting reactions.

## Materials and methods

### Experimental section

#### Preparation of COK-47 and COK-47-bpy

The synthesis of COK-47 was conducted inside a 30 mL microwave vial. First, 242 mg of biphenyl-4,4′-dicarboxylic acid (H_2_bpdc) (1 mmol) were suspended in a volumetric 1 : 9 mixture of HPLC-grade methanol and absolute dimethylformamide. The vial was flushed with argon and the suspension sonicated for 10 minutes. Afterwards, 148 μL (0.5 mmol) of titanium tetraisopropoxide (TTIP) were added under an argon atmosphere. The vial was then closed and the suspension stirred for 2–3 minutes. Subsequently, it was heated to 150 °C using microwave irradiation and held at that temperature for one hour under magnetic stirring. After cooling down to 55 °C, the product was separated from the reaction medium by vacuum filtration and washed each with 90 mL DMF and 90 mL methanol. The filtered product was pre-dried under a vacuum at 100 °C for 30 m. Subsequently, it was milled and dried under a vacuum at 150 °C overnight. COK-47-bpy was synthesized following the same procedure with 1 mmol (244 mg) of 2,2′-bipyridine-5,5′-dicarboxylic acid (H_2_bpydc) was used as a linker instead. COK-47 and COK-47-bpy was characterized using XRD, FTIR and and UV-Vis spectroscopy. All characterizations match well with reported literature (Fig. S6†).

#### Preparation of MIL-125

MIL-125 was prepared *via* solvothermal synthesis according to a previous report.^43^ Briefly, 1 g (6 mmol) terephthalic acid was added to a solution mixture of 2 mL HPLC-grade methanol and 18 mL absolute DMF in a Teflon-lined autoclave, the reactor was then thoroughly flushed with argon. Next, the terephthalic acid suspension was allowed to stir for 50 minutes followed by the addition of 568 μL TTIP (1.9 mmol) under a constant argon atmosphere and magnetic stirring. The reaction mixture was sonicated for 3 minutes and additionally stirred for 30 minutes. It was then heated up to 150 °C in the autoclave for 18 hours. After naturally cooling down to room temperature, 60 mL DMF was added to the mixture and it was sonicated for 8 minutes. The product was separated *via* vacuum filtration and washed 3 times with 20 mL methanol respectively, followed by vacuum drying at 150 °C. Once dried MIL-125 was characterized using XRD, FTIR and UV-Vis spectroscopy (Fig. S7†).

### Characterization methods of COK-47

To evaluate the quality and purity of the synthesized catalysts, Fourier-transformation infrared spectroscopy and powder-X-ray diffractometry were used. Infrared spectra (IR) were taken with a PerkinElmer UATR Two FT-IR spectrometer by pressing the sample powder onto the ATR-crystal. The spectra were taken from 400 cm^−1^ to 4000 cm^−1^. Powder-X-ray diffractometry (XRD) was carried out at a PANalytical X'Pert Pro multi-purpose diffractometer with a Si single crystal as a sample carrier. TGA measurements were carried out using TGA8000 from PerkinElmer with a program set to heat at the rate of 5 °C m^−1^. Optical properties were analyzed *via* diffuse reflectance spectroscopy (DRS) on the synthesized catalysts to determine the energy of their band gaps according to the method described by Makuła *et al.*^44^ These measurements were taken with the Jasco V-670 UV-Vis photo spectrometer, with MgSO_4_ as a background. Zeta potential measurements were conducted with a Malvern Zetasizer Nano ZSP. X-ray fluorescence spectroscopy (XRF) was done using an Atomika 8030C X-ray fluorescence analyzer (Atomika Instruments GmbH, Oberschleissheim, Munich, Germany) in total reflection geometry (T-XRF) with a molybdenum X-ray source (monochromatized Kα-line). The excitation conditions were 50 kV and 47 mA for 100 s irradiation time and an energy-dispersive Si(Li)-detector was employed. X-ray photoelectron spectroscopy (XPS) was acquired on a custom-built SPECS XPS-spectrometer consisting of a monochromatised Al-Kα X-ray source (μFocus 350) and a hemispherical WAL-150 analyser. Each sample was mounted onto a holder using double-sided Cu/carbon tape. The samples were measured under the following conditions: using an excitation energy, 1486.6 eV; beam energy and spot size was 70 W on 400 μm; with an angle of 51° to sample (surface normal) and the base pressure of 4 × 10^−10^ mbar with the pressure of 7 × 10^−10^ mbar during measurements.

### Standard curve and figure of merit

To accurately measure the dye concentration in the solution, a standard curve was drawn by measuring the absorption intensities of a set of MB solutions with known concentrations in the range of 0.5 to 10 ppm. To maintain homogeneity, each of these solutions was prepared by diluting a 50 ppm MB stock solution. A linear regression fit was made from the measured data points providing a standard curve equation that was used to estimate the MB concentration for all the photodegradation experiments. The same method was used for CV, TB and MO as well. (Additional details in ESI†).

Secondly, in an attempt to compare and evaluate the overall performance of a photocatalyst, the overall removal efficiency is used as an important figure of merit.^28^ The overall removal efficiency is calculated according to the formula,
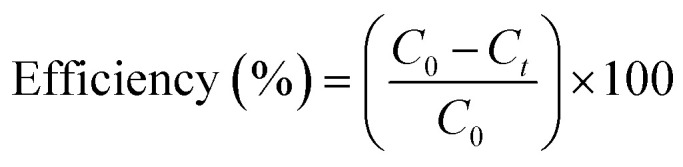
where, *C*_0_ and *C*_*t*_ are the MB concentration right before the illumination and at any time *t* during illumination, respectively.^45^

### Rate calculations

For the calculation of the kinetic constants of the degradations, it was assumed that the degradation followed the kinetics of a reaction of either first, second, or third order, depending on the shape of the degradation curve.

Reactions of the first order can be described using the following equation.*C*_*t*_ = *C*_0_ × e^−*kt*^here, the kinetic constant *k* was then calculated by plotting ln(*C*_0_/*C*_*t*_) against time (in seconds) and fitting a linear function with a fixed *y*-intercept at 0 to the data points.^46^

Reactions of second order can be described using the following equation.
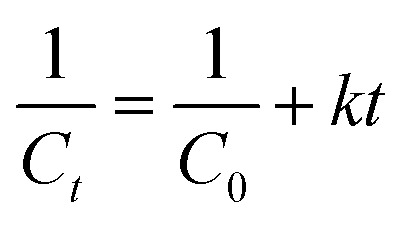


For those reactions, the kinetic constant was calculated by plotting 
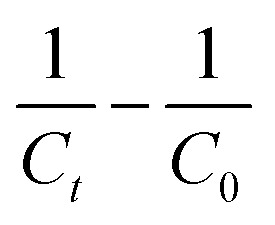
 (*C* in mol L^−1^) against the reaction time (in seconds). Reactions of third order can be described with the following equation.
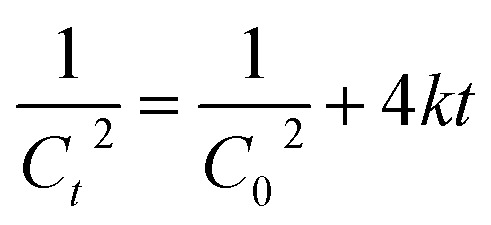


For those reactions, the kinetic constant was calculated by plotting 
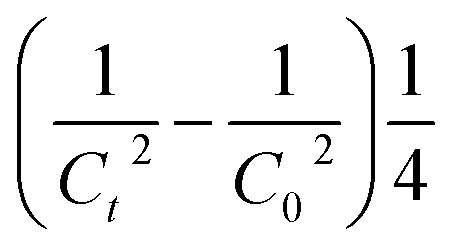
 (*C* in mol L^−1^) against the reaction time (in seconds).

### Mixed dye adsorption studies

In a single experiment, 25 mL solution of 50 ppm methylene blue was mixed with 25 mL of 50 ppm methyl orange and stirred for 30 min. After mixing, the solution was filtered through a filter paper in order to remove formed particles, yielding a clear greenish (∼6 ppm) MB/MO solution. Next, 25 mL of this solution was mixed with 25 mL of pre-sonicated COK-47 suspension in water (0.2 mg mL^−1^). The first sample for UV-Vis analysis was taken 30 s after mixing, followed by sampling at 5, 15, 30, 60, 90, and 120 min. Each sample was centrifuged at 5600 rpm for 20 min, followed by UV-Vis measurement to determine the dye concentration.

### Photodegradation of MB

Each experiment was conducted in a homemade slurry-type reactor equipped with a water-cooling jacket. A 200 W lamp (Lumatec SUV-DC-E) was used for illumination with a fixed illumination distance of 8.6 cm with an intensity of 32.8 mW cm^−2^ for visible light (400–700 nm) and 17.2 mW cm^−2^ for UV-light (240–400 nm). Briefly, 10 mg of the pristine catalyst was suspended in 25 mL of deionized water and sonicated for 15 minutes to obtain a homogeneous suspension. This suspension was then transferred into the reactor and mixed with 25 mL of 50 ppm dye solution, leading to an overall methylene blue concentration of 25 ppm in the final solution. After mixing, immediately, the first sample of 2 mL was taken, followed by an “equilibration phase” of stirring in the dark for two hours to reach an adsorption-maximum. Following this, another 2 mL sample was taken and illumination was started. During the “illumination phase”, 2 mL samples were taken after 5, 15, 30, 60, 90, and 120 minutes of illumination. The solution was then separated from the catalyst by centrifugation and the concentration of methylene blue was measured using UV-Vis spectroscopy. The characteristic peak intensities of methylene blue (MB), crystal violet (CV), and toluidine blue (TB) at 664, 590, and 630 nm, respectively were used to evaluate the photocatalytic degradation performance of each of the materials. The reaction temperature was maintained at 15 °C throughout the experiment under constant stirring of 200 rpm.

### PZC measurements

To determine the point-of-zero charge 0.1 M KNO_3_ solutions, adjusted to 5 pH points using 0.1 M HCl and 0.1 M KOH, measured with a pH meter. Briefly, 10 mg of MOF powder was dispersed in 15 solution *via* ultrasonication for 10 min. The homogeneous suspensions were stirred for 24 h at 500 rpm under dark. Following this, 10 mL of the solution was centrifuged at 5600 rpm for 15 min. The pH of the supernatant was determined using a pH meter. The difference between the initial and the final pH of the solution was plotted against the initial pH to determine the point-of-zero charge.

### Zeta potential measurements

Zeta potential measurements were carried out using water as a solvent. Briefly, 10 mg of powder was suspended in de-ionised water *via* ultrasonication for 10 min. Each measurement consisted of ∼12 to 15 runs to obtain a stable value that is in good agreement with the model and had a standard deviation of less than 10%.

### Sunlight-mediated photo-degradation experiments

The suspensions for the sunlight experiments were prepared similarly to the standard setup (10 mg catalyst in 50 mL 25 ppm methylene blue solution). Instead of in the cooled reactor, to simulate/mimic real-world scenarios, the experiments were performed in beakers, under magnetic stirring. The average intensity of the natural sunlight was measured to be 11 mW cm^−2^.

### Photoelectric test setup

#### Electrode preparation

Briefly, FTO glass electrodes (∼3 × 1 cm^2^) were ultrasonicated for 15 min each in soap solution, DI water, and finally with EtOH, to obtain clean surfaces for drop casting. These electrodes were then dried at 60 °C and masked at top 1 × 1 cm using Kapton tape, 30 min before use. Simultaneously, 10 mg of catalyst powder was dispersed in 200 μL of isopropanol solution *via* ultrasonication for 10 min. Following this, ∼80 μL of this homogeneous suspension was drop-casted onto the masked FTO electrodes and allowed to dry at RT followed by drying at 60 °C for 30 min. The total electrode area covered with catalyst material was 2 × 1 cm^2^.

#### Chopped light-chronoamperometry tests

Chopped light-chronoamperometry tests were carried out in a single gas-tight cell using catalyst on FTO glass, Pt plate, and Ag/AgCl (in 3 M KCl) as the working, counter, and reference electrode respectively, dipped in 0.1 M K_2_SO_4_ electrolyte solution. The solution was purged with N_2_ for 1 h before testing. Chronoamperometry tests were done at 0 V *vs.* OCP with light on–off cycles with either an illumination ranging from 280–400 nm (UV) or using 400–700 nm (visible) in the case of each sample. Each illumination cycle included light on for about 60 s followed by 15 s of no illumination.

## Data availability

Data are available upon request from the authors.

## Author contributions

Conceptualization, SNM, ML, DE and AC; methodology, SNM, ML and AC; investigation, SNM, ML, HR, SB, MSE and PA; resources, AC, DE; data curation, SNM and ML; writing—original draft preparation, SNM, ML, and AC; writing—review and editing, SNM, ML, HR, SB, PA, MSE, AC and DE; visualization, SNM; supervision, AC and DE; project administration, AC; funding acquisition, AC. All authors have read and agreed to the published version of the manuscript.

## Conflicts of interest

There are no conflicts to declare.

## Supplementary Material

TA-012-D4TA01967A-s001
