# Supplementary material for: Harnessing a Ti-based MOF for selective adsorption and visible-light-driven water remediation
Source: J Mater Chem A Mater. 2024 Jun 4;12(31):19924–34. doi: 10.1039/d4ta01967a (PMC11302510; doi:10.1039/d4ta01967a)
Supplement: TA-012-D4TA01967A-s001 [file TA-012-D4TA01967A-s001.pdf]

Electronic Supplementary Material (ESI) for Journal of Materials Chemistry A.
This journal is © The Royal Society of Chemistry 2024

Electronic Supplementary Information

Harnessing a Ti-based MOF for selective adsorption and visible-light-driven water remediation

Stephen Nagaraju Myakala[a], Magdalena Ladisich[a], Pablo Ayala[a], Hannah Rabl[a], Samar Batool[a], Michael S. Elsaesser[b], Alexey Cherevan[a], Dominik Eder[a]**

[a]*TU Wien, Institute of Materials Chemistry, Division Molecular Materials Chemistry, Getreidemarkt 9/BC/02, 1040, Vienna, Austria*

[b]*Paris-Lodron-University of Salzburg, Department Chemistry and Physics of Materials, 5020, Salzburg, Austria*

Corresponding authors: alexey.cherevan@tuwien.ac.at, dominik.eder@tuwien.ac.at

Contents

Pristine COK-47 characterization 3

Supplementary note 1 | TGA Analysis 4

Surface Analysis 4

Table S1 | Overview of the adsorption capacity (Q_e) values for a variety of MOFs reported previously towards dye adsorption in comparison to current work. 4

Supplementary note 2 | Adsorption studies 5

Reference measurements 5

Supplementary note 3 | Photobleaching experiments 6

Supplementary note 4 | Sunlight experiments 6

Monomer vs dimer adsorption 6

Supplementary note 5 | Scavengers reference experiments 7

Supplementary note 6 | Chopped light photoelectric measurements 8

Post catalytic analysis 8

Reference materials | COK-47-bpy, MIL-125-Ti 9

Pristine COK-47 characterization

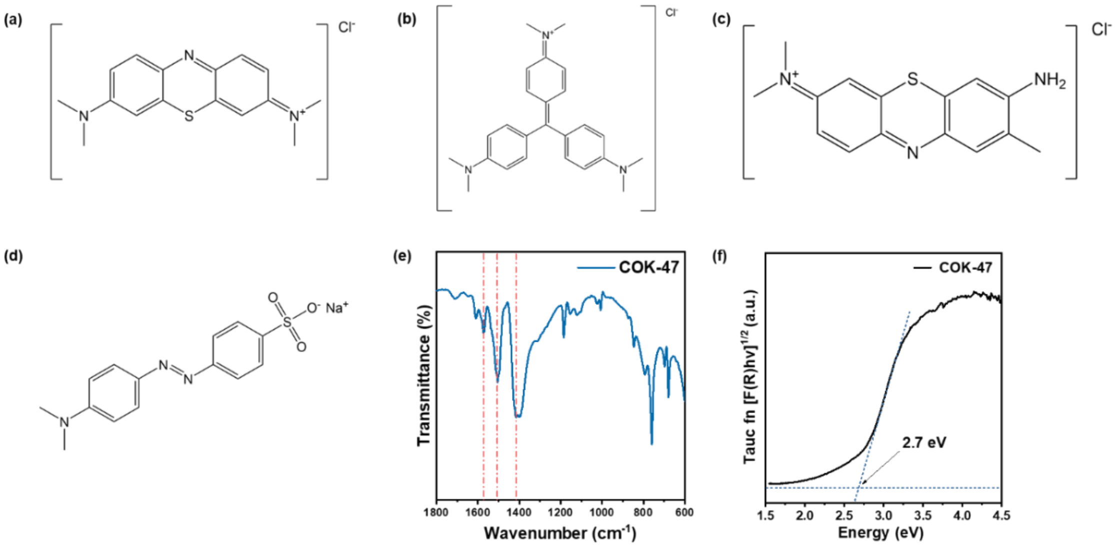

Figure S1 | Structural representations of **(a)** methylene blue, MB **(b)** crystal violet, CV **(c)** toluidine blue, TB and **(d)** methyl orange, MO; **(e)** FTIR spectrum of pristine COK-47 showing the characteristic COO^- peaks indicated with 3 dotted lines and **(f)** tauc-function plot (assuming a direct band-gap) of pristine COK-47 powder.

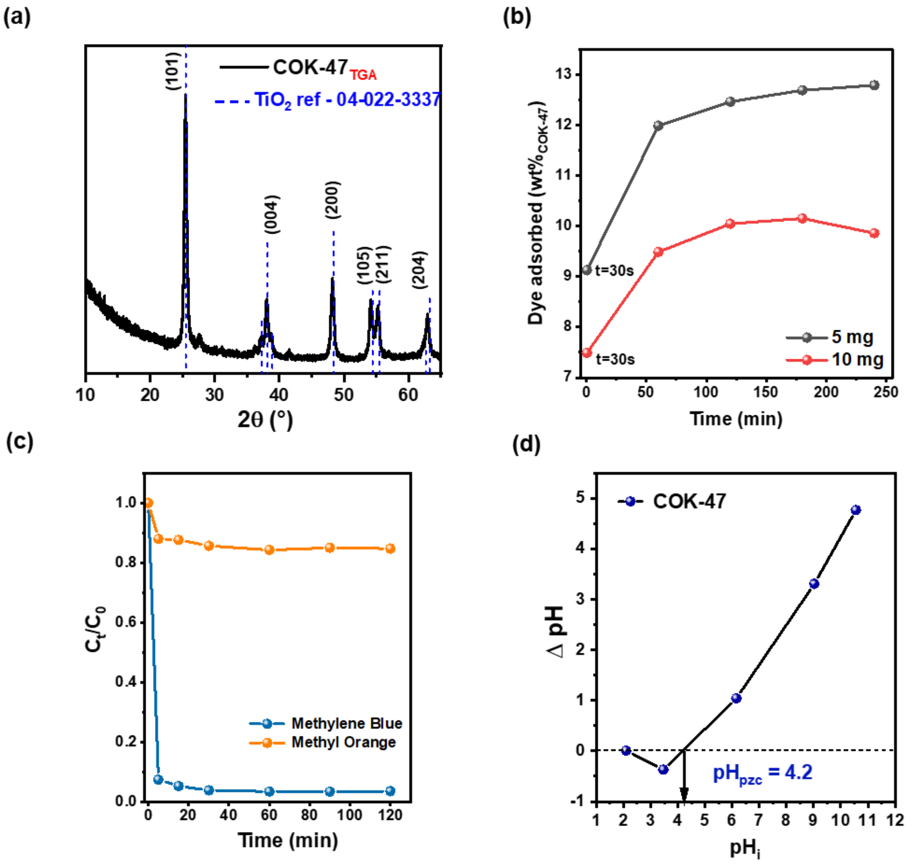

Figure S2 | (a) XRD pattern of COK-47 after TGA measurement (heated until 700°C) in synthetic air atmosphere with blue lines corresponding to the expected TiO_2 pattern (04-022-3337) **(b)** methylene blue adsorption studies using two adsorbent (COK-47) dosages **(c)** Dye adsorption curves when using COK-47 in a mixture of methylene blue and methyl orange solutions (6 ppm) after equilibration (120 min) **(d)** PZC curves indicating the isoelectric point for pristine COK-47.

Supplementary note 1 | TGA Analysis

First, we calculate the molecular weight of COK-47 assuming the formula $Ti_2O_3(C_{14}H_8O_4)$, taken from literature.[1] This calculation gives a value of 383.94 g mol^{-1}. Since we know the final product of COK-47 oxidative decomposition to be TiO_2 (evident from Figure S2), we can calculate the theoretical weight loss, assuming a perfect COK-47 structure. Based on Equation 1, we estimate the total weight loss of the reaction to be ~58 wt% after the full conversion into TiO_2 occurs. TGA curves obtained for pristine COK-47 (Figure 1c of the main text) show an overall weight loss of ~60% (in air) – a value that is higher than 58 wt% expected for the stoichiometric structure. This implies the presence of excess ligands, which we believe could be present at the surface of COK-47 nanocrystals as surface passivation (as illustrated in Figure 1b of the main text), thus effectively accounting for the higher than expected ligand:Ti ratio.

$$Ti_2O_3(C_{14}H_8O_4) \xrightarrow{650°C} TiO_2 \qquad (1)$$

Surface Analysis

Table S1 | Overview of the adsorption capacity (Q_e) values for a variety of MOFs reported previously towards dye adsorption in comparison to current work.

MOF	Pollutant	Q_e (mg g^{-1})	Equilibrium time	Pollutant concentration (ppm)
**COK-47**[This work]	MB	100.5	60 min	25
**UiO-66-Zr**[3]	MB	81	200 min	50
**Zr-BDC-CP**[4]	MB	39	5h	25
**MIL-100(Fe)**[5]	MB	736.2	-	60
**MIL-100(Cr)**[5]	MB	645.3	-	25
**HKUST-1**	MB	123	-	50
**MOF-235**[6]	MB	187	80 min	25
**Fe-BDC**[7]	MB	8.65	90 min	5
**UiO-66-Zr**[8]	AR	400	90 min	50
**Cu-BTC**[9]	MB+MO+RhB	39.5	30 min	100
**$Ni(BIC)_2.2.5H_2O$**[10]	MB	15.5	60 min	5
**MIL-53(Al)-NH_2**[11]	MB/MG	45/37.8	60 min	5
**MIL-53(Al)**[11]	MB/MG	3.6/2.9	60 min	5

AR: Alizarin red; MB: Methylene blue; RhB: Rhodamine B; MG: malachite green.

Supplementary note 2 | Adsorption studies

Based on relevant literature, we assume the (interaction/adsorption) surface area (i.e. footprint) of a single methylene blue molecule to be ~ 17 x 7.6 Å^2 .[2] Based on the experimentally reported surface area of COK-47 (~285 m^2 g^{-1}) and assuming a monolayer adsorption model, one can estimate a monolayer MB coverage to correspond to ~2.2 x 10^{20} dye molecules per gram of MOF. This number of MB molecules can be translated to ~366 µmol or 117 mg of MB per gram of COK-47, corresponding to ~12 wt% of its weight.

Experimental adsorption tests were carried out using 10 ml of 25 ppm of MB solution and 2 mg of COK-47. The powder was homogeneously suspended in the MB solution via ultrasonication for 15 min followed by stirring the suspension for 2 h under dark. The suspension was then centrifuged at 5000 rpm for 15 min, followed by measuring the UV-Vis spectrum of the supernatant solution to quantify the remaining dye in the solution. We observed a strong decrease from 25 ppm to ~5.3 ppm, corresponding to an uptake of ~0.2 mg MB, thereby revealing an adsorption capacity of 100 mg g^{-1} or ~ 10 wt% of the COK-47 mass, which is in good agreement with the theoretical maximum estimated above.

Reference measurements

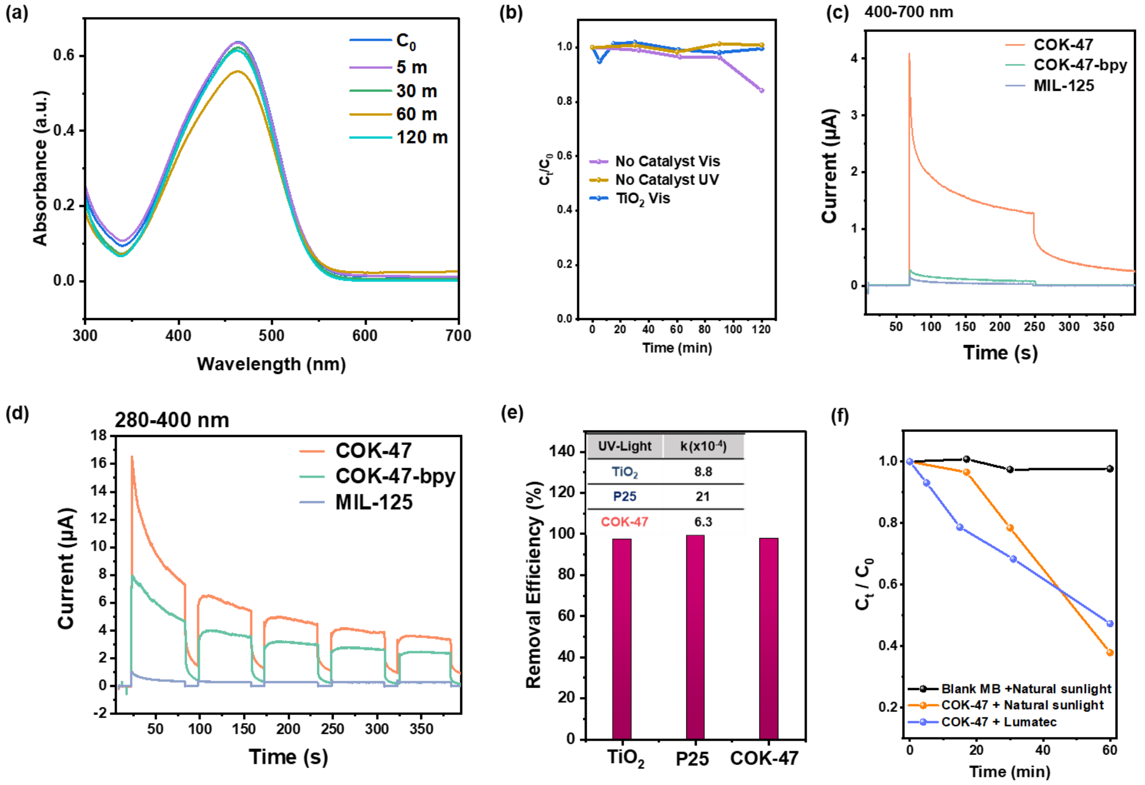

Figure S3 | (a) UV-Vis spectrum of methyl orange on COK-47 under visible light illumination over a period of 2 h **(b)** Concentration profiles of methylene blue over a period of 2 h under UV and visible light illumination without the catalyst, and with (Anatase) TiO_2 under visible light illumination **(c)** transient photocurrent response of COK-47, COK-47-bpy, and MIL-125-Ti under visible light and **(d)** UV illumination; **(e)** overall MB removal efficiency of COK-47, P25 (commercial TiO_2) and TiO_2 (Anatase) under UV (280-400 nm) illumination after 2 h and **(f)** concentration profiles of methylene blue with and without COK-47 in the presence of natural sunlight, and artificially-derived visible light

Supplementary note 3 | Photobleaching experiments

To completely rule out unknown degradation effects caused by the illumination on the pristine dye molecules in the solution, we measured the concentration profile of aqueous methylene blue over the span of 2 h in the absence of any catalyst powder. Figure S3a clearly shows that there are no changes in the dye concentration thereby ruling out any self-degradation in the presence of light.

Supplementary note 4 | Sunlight experiments

Methylene blue degradation profiles in Figure S3f show that the performance of COK-47 under natural sunlight is similar to that under simulated visible light (400-700 nm, Lumatec) despite the much lower intensity of the natural sunlight (~11 mW cm^{-2} vs 32.8 mW cm^{-2} of the artificial light source). We suggest that the degradation rate achieved under solar light is made of two additional contributions compared to that under visible (400-700 nm) light: facilitated photodegradation due to high-energy UV portion (2-5 % of the solar light spectrum) and natural thermal heating due to the IR portion of the photons, which further enhances the process. Hence, the overall degradation performance under solar light seems on par with that of lab simulated visible-light illumination conditions.

Monomer vs dimer adsorption

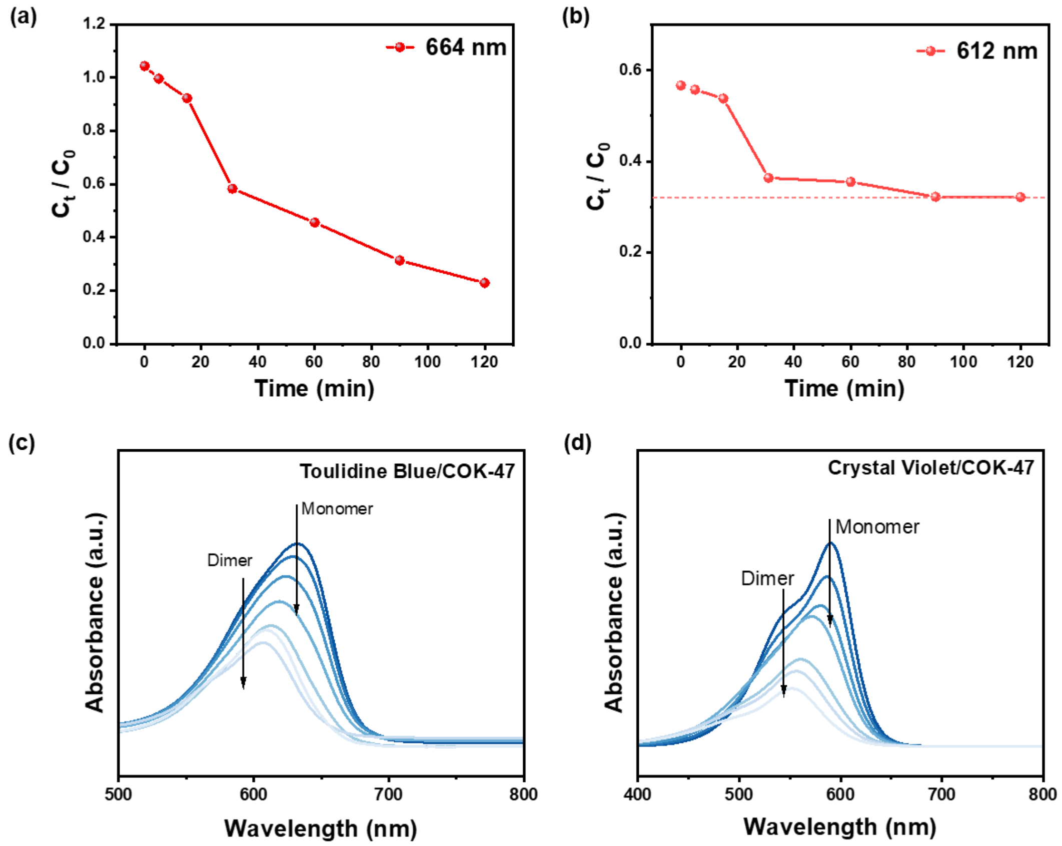

Figure S4 | MB concentration profiles considering **(a)** monomeric (MB_1) and **(b)** dimeric (MB_2) form of methylene blue at 664 and 612 nm, respectively, under photodegradation conditions; UV-vis absorption profiles showing the degradation of monomeric and dimeric forms of **(c)** toluidine blue, TB, and **(d)** crystal violet, CV, using COK-47 under visible light illumination

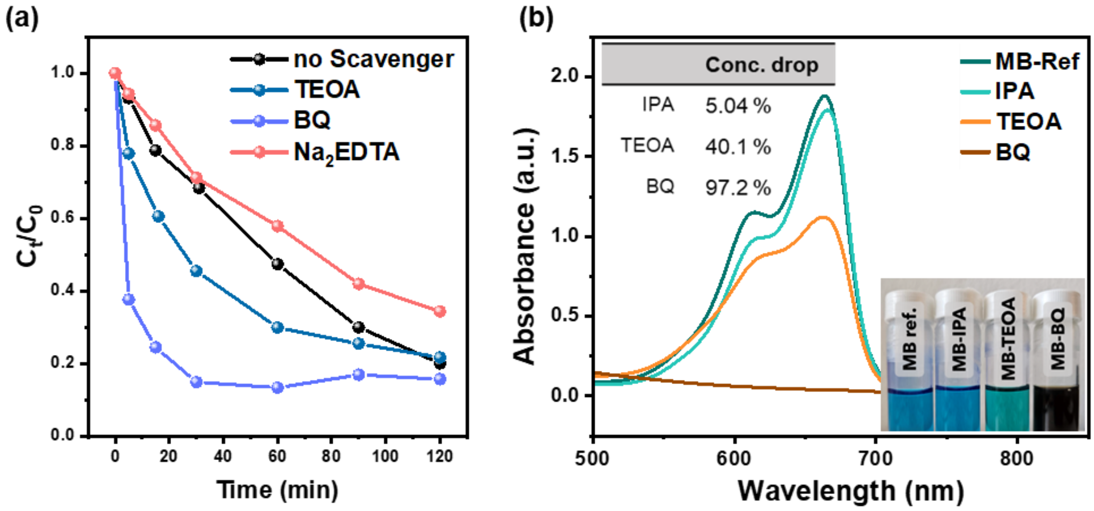

Figure S5 | (a) MB concentration profiles of photodegradation experiments using COK-47 in the presence of different scavengers such as BQ, TEOA, and Na_2EDTA; **(b)** UV-Vis absorption profiles of MB after photochemical reaction with BQ and TEOA along with insets showing the amount of MB degradation (expressed as concentration drop) and the photographs of the MB/scavenger solutions after illumination.

Supplementary note 5 | Scavengers reference experiments

As stated in the main manuscript, surprisingly we observed a ~1.5 and ~2.4 fold increase in the MB degradation rates when our suspensions were irradiated in the presence of BQ and TEOA, respectively (Figure S5a). In the case of BQ which typically acts as $O_2^{\cdot -}$ scavenger, we believe that one of the intermediates of this reaction (such as $BQ^{\cdot -}$ radical) can be responsible for activating another oxidation pathway, this not only leads to MB degradation but also favors the $O_2^{\cdot -}$ radical formation thereby increasing charge separation, leading to an overall increased MB degradation rate. Similarly, the increased degradation rates in the presence of TEOA – which acts as a direct hole scavenger at the solution-MOF interface – can also be explained by considering overall improved charge separation via efficient electron extraction from the MOF. We thus propose that efficient hole extraction by TEOA results in long-lived electrons capable to reduce atmospheric O_2 molecules present in the solution indicating that so-formed $O_2^{\cdot -}$ radicals are relevant to the MB degradation process. In the presence of Na_2EDTA (Figure S5a), while we observe a drop in the degradation rate (from $22.1*10^{-5}$ to $6.7*10^{-5}$), our reference experiments in the absence of catalyst also indicate that MB degradation takes places already due inherent chemical reaction between MB and Na_2EDTA in the presence of light. Similarly, our reference studies using BQ and TEOA scavengers in the absence of any catalyst revealed degradation of the dye molecules in the presence of light (Figure S5b, inset), which can be at least to some extent related to the acceleration of MB degradation observed in our photocatalytic tests. In contrast to this, IPA and $CHCl_3$ did not show such behaviour.

Supplementary note 6 | Chopped light photoelectric measurements

Figure S3c reveals that COK-47-bpy generates a photoresponse twice lower than its pristine counterpart, which could be the result of charge trapping nature of its pyridine-containing ligands that hinder charge extraction and utilization. Additionally, despite having excellent absorption in the UV, MIL-125-Ti does not give high photocurrent response most likely due to low charge mobility due to its 0D isolated SBUs in the structure as opposed to the 2D-SBU based COK-47.[12]

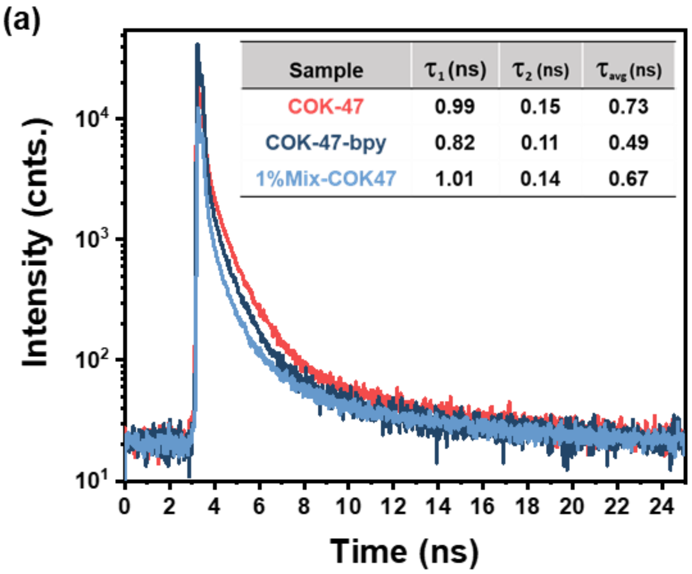

Figure S6 | (a) Time-resolved emission spectra of pristine COK-47 (orange), COK-47-bpy (dark blue), and 1mol% BPyDC COK-47 (pale blue).

Post catalytic analysis

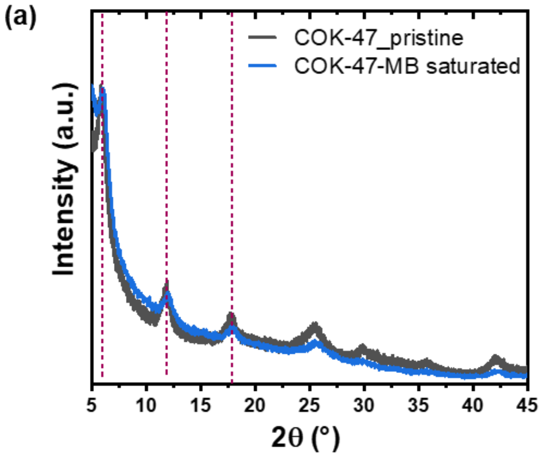

Figure S7 | (a) XRD pattern of pristine COK-47 and MB-saturated COK-47 showing no structural changes of the MOF. Red lines indicate the characteristic peak positions of COK-47 extracted from previous reports.[1]

Reference materials | COK-47-bpy, MIL-125-Ti

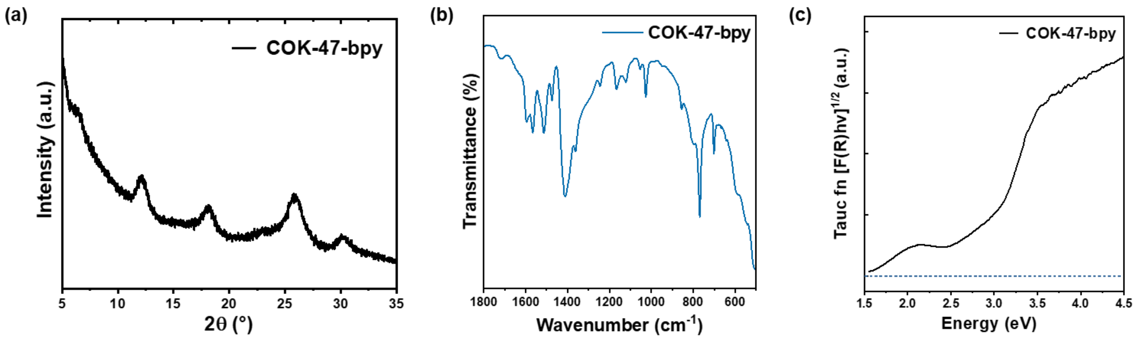

Figure S8 | (a) XRD pattern of pristine COK-47-bpy, **(b)** FTIR spectrum of COK-47-bpy with all characteristic peaks corresponding to COO^- and **(c)** DRS (UV-Vis reflectance) spectrum of pristine COK-47-bpy powder.

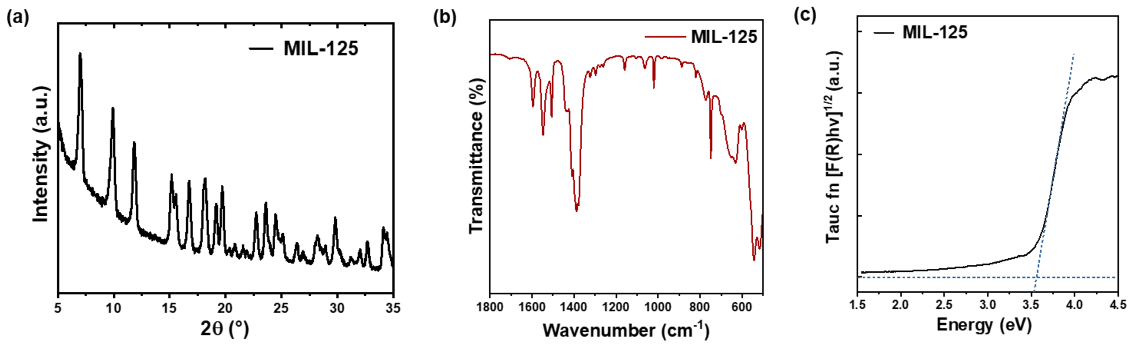

Figure S9 | (a) XRD pattern of pristine MIL-125-Ti, **(b)** FTIR spectrum of MIL-125-Ti featuring all characteristic peaks and **(c)** DRS (UV-Vis reflectance) spectrum of pristine MIL-125-Ti powder.

References

(1) Ayala, P.; Naghdi, S.; Nandan, S. P.; Myakala, S. N.; Rath, J.; Saito, H.; Guggenberger, P.; Lakhanlal, L.; Kleitz, F.; Toroker, M. C.; Cherevan, A.; Eder, D. The Emergence of 2D Building Units in Metal-Organic Frameworks for Photocatalytic Hydrogen Evolution: A Case Study with COK-47. *Adv. Energy Mater. n/a* (n/a), 2300961. https://doi.org/10.1002/aenm.202300961.

(2) Arias, M.; López, E.; Nuñez, A.; Rubinos, D.; Soto, B.; Barral, M. T.; Díaz-Fierros, F. Adsorption of Methylene Blue by Red Mud, An Oxide- Rich Byproduct of Bauxite Refining. In *Effect of Mineral-Organic-Microorganism Interactions on Soil and Freshwater Environments*; Berthelin, J., Huang, P. M., Bollag, J.-M., Andreux, F., Eds.; Springer US: Boston, MA, 1999; pp 361–365. https://doi.org/10.1007/978-1-4615-4683-2_39.

(3) *Aqueous Synthesis of a Mesoporous Zr-Based Coordination Polymer for Removal of Organic Dyes | ACS Omega*. https://pubs.acs.org/doi/10.1021/acsomega.9b03192 (accessed 2024-02-14).

(4) Mohammadi, A. A.; Alinejad, A.; Kamarehie, B.; Javan, S.; Ghaderpoury, A.; Ahmadpour, M.; Ghaderpoori, M. Metal-Organic Framework Uio-66 for Adsorption of Methylene Blue Dye from Aqueous Solutions. *Int. J. Environ. Sci. Technol.* **2017**, *14* (9), 1959–1968. https://doi.org/10.1007/s13762-017-1289-z.

(5) Tong, M.; Liu, D.; Yang, Q.; Devautour-Vinot, S.; Maurin, G.; Zhong, C. Influence of Framework Metal Ions on the Dye Capture Behavior of MIL-100 (Fe, Cr) MOF Type Solids. *J. Mater. Chem. A* **2013**, *1* (30), 8534–8537. https://doi.org/10.1039/C3TA11807J.

(6) Haque, E.; Jun, J. W.; Jhung, S. H. Adsorptive Removal of Methyl Orange and Methylene Blue from Aqueous Solution with a Metal-Organic Framework Material, Iron Terephthalate (MOF-235). *J. Hazard. Mater.* **2011**, *185* (1), 507–511. https://doi.org/10.1016/j.jhazmat.2010.09.035.

(7) Arora, C.; Soni, S.; Sahu, S.; Mittal, J.; Kumar, P.; Bajpai, P. K. Iron Based Metal Organic Framework for Efficient Removal of Methylene Blue Dye from Industrial Waste. *J. Mol. Liq.* **2019**, *284*, 343–352. https://doi.org/10.1016/j.molliq.2019.04.012.

(8) Embaby, M. S.; Elwany, S. D.; Setyaningsih, W.; Saber, M. R. The Adsorptive Properties of UiO-66 towards Organic Dyes: A Record Adsorption Capacity for the Anionic Dye Alizarin Red S. *Chin. J. Chem. Eng.* **2018**, *26* (4), 731–739. https://doi.org/10.1016/j.cjche.2017.07.014.

(9) Li, Y.; Gao, C.; Jiao, J.; Cui, J.; Li, Z.; Song, Q. Selective Adsorption of Metal–Organic Framework toward Methylene Blue: Behavior and Mechanism. *ACS Omega* **2021**, *6* (49), 33961–33968. https://doi.org/10.1021/acsomega.1c05299.

(10) Shi, L.; Hu, L.; Zheng, J.; Zhang, M.; Xu, J. Adsorptive Removal of Methylene Blue from Aqueous Solution Using a Ni-Metal Organic Framework Material. *J. Dispers. Sci. Technol.* **2016**, *37* (8), 1226–1231. https://doi.org/10.1080/01932691.2015.1050731.

(11) Li, C.; Xiong, Z.; Zhang, J.; Wu, C. The Strengthening Role of the Amino Group in Metal–Organic Framework MIL-53 (Al) for Methylene Blue and Malachite Green Dye Adsorption. *J. Chem. Eng. Data* **2015**, *60* (11), 3414–3422. https://doi.org/10.1021/acs.jced.5b00692.

(12) Abdul Mubarak, N. S.; Foo, K. Y.; Schneider, R.; Abdelhameed, R. M.; Sabar, S. The Chemistry of MIL-125 Based Materials: Structure, Synthesis, Modification Strategies and Photocatalytic Applications. *J. Environ. Chem. Eng.* **2022**, *10* (1), 106883. https://doi.org/10.1016/j.jece.2021.106883.
